# Ecological dark matter: Insect decline and the planetary monitoring gap

**DOI:** 10.1371/journal.pbio.3003941

**Published:** 2026-08-18

**Authors:** Justin Crocker

**Affiliations:** European Molecular Biology Laboratory, Heidelberg, Germany

## Abstract

Wherever we look, insect numbers are declining, but most insect life on Earth is found in locations we are not actively monitoring. This Perspective discusses the insect decline and how can we build in the next decade the planetary observatory that biodiversity requires.

## Teaser

Wherever we look, insect numbers are declining, but most insect life on Earth is found in locations we are not actively monitoring. The defining task of the next decade will be to build the planetary observatory that biodiversity science requires.

“We are accustomed to look for the gross and immediate effect and to ignore all else”—*Rachel Carson, Silent Spring (1962)*.

Insects are not one charismatic taxon among many; they are the load-bearing layer of terrestrial ecosystems, pollinating most flowering plants, cycling dead organic matter, and forming the base of the food webs that vertebrates depend on. A sustained loss of insect abundance is therefore not the loss of one group but a structural change in how terrestrial ecosystems function. Reports of an ‘insect apocalypse’ have shaped a decade of public debate, but the primary literature supports a more constrained, yet more consequential, conclusion.

**Fig 1:**
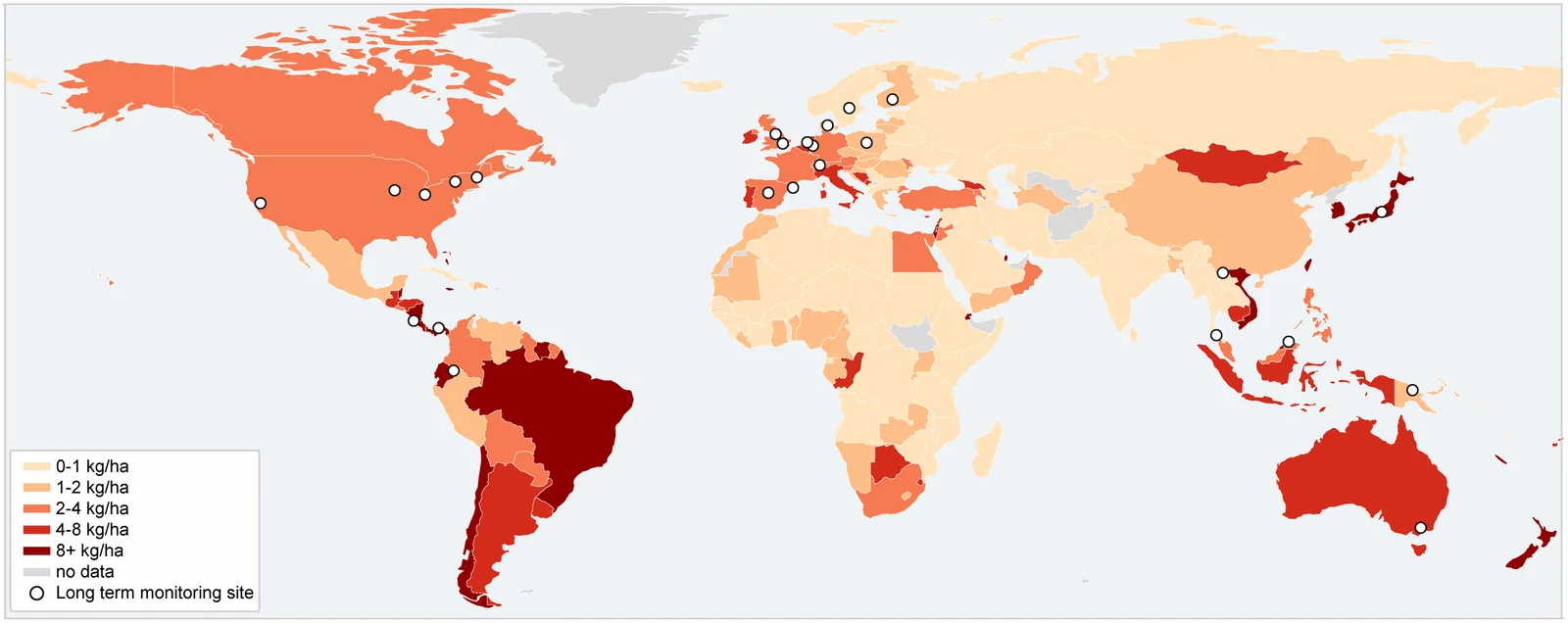
Long-term insect monitoring sites and global pesticide use intensity. Total pesticide use per area of cropland (kg ha^−1^), most recent year available per country (2015–2023), from the FAOSTAT Pesticides Use domain; gray indicates no reported data. FAOSTAT totals exclude seed-applied systemic pesticides and so understate exposure to the neonicotinoids emphasized in the text. White points mark representative long-term insect monitoring programs. Base map: Natural Earth, (public domain; https://naciscdn.org/naturalearth/110m/cultural/ne_110m_admin_0_countries.zip).

Long-term declines in terrestrial insect abundance and biomass are robustly documented across well-monitored regions, especially in North America and, less consistently, temperate Europe. For example, a German Malaise-trap dataset showed a 76% seasonal decline in flying insect biomass over 27 years [1]. Global syntheses estimate average terrestrial declines of roughly 9% per decade, although the magnitude, and especially reports of rising freshwater abundance, has been contested, and trends vary substantially among taxa and habitats [2]. The evidence does not show that all insects are collapsing everywhere; what it does shows is more specific, and more troubling. Where careful long-term monitoring exists, decline has been the dominant terrestrial signal, and that monitoring barely extends beyond the temperate zone. That gap is not a neutral blank to be read as stability; it coincides with the regions where the documented drivers of decline are intensifying the fastest (Fig 1).

The paradox is that the best-monitored regions are not those where most insect life exists. Despite holding most of the world’s insect diversity, the tropics contribute only a small fraction of long-term monitoring datasets (Fig 1); standardized time series remain concentrated in temperate Europe and North America, which hold only a small fraction of global insect species [3]. Most insect diversity therefore exists as a form of ecological dark matter: biologically central, massively abundant, and systematically undermeasured.

A lack of observation is not the same as an absence of change, and much of the unmeasured majority is exposed to the same drivers. Climate change reaches even intact tropical forests, while chemical exposure concentrates along agricultural frontiers; with roughly half of tropical forest already cleared or converted to agriculture [3], that frontier and the diversity it threatens share the same ground. Tropical insects also sit closer to their thermal optima, with narrower safety margins, so equivalent warming should have disproportionately larger effects at low latitudes, not smaller [4]. This asymmetry distorts attribution as well as accounting. Habitat loss, agricultural intensification, pesticide exposure, climate warming, light pollution, and nitrogen deposition are all implicated in specific systems, but few landscapes have been monitored deeply enough to quantify their relative contributions [5]. Decline is unlikely to stem from single stressors acting independently: raised temperatures amplify pesticide toxicity in the laboratory; fragmented landscapes plausibly slow recovery; and chemical and climatic pressures are expected to interact nonlinearly.

Among individual drivers, pesticides currently have some of the most direct supporting evidence, although it rests on a narrow base and the field-scale signal remains correlational. Large-scale butterfly analyses in the Midwest USA reveal insecticides, particularly neonicotinoid seed treatments, as among the strongest measured correlates of decline [6]. Pesticides are highlighted not as the presumed dominant driver but because exposure is directly quantifiable and transparent reporting could sharply improve attribution. A recent screen of more than a thousand agrochemicals at environmentally realistic concentrations found widespread sublethal behavioral and developmental effects in fruit flies and additional insects, amplified by heat and partly conserved across taxa [7]. Sublethal effects in a laboratory do not, on their own, establish population-level decline (exposure, mixtures, and timing all intervene), but they do highlight a hazard that the field correlations implicate. Because agrochemical use is large and expanding along lower-latitude frontiers, the warm regions we do not monitor are precisely where exposure-related effects should be strongest, not absent (Fig 1), yet long-term monitoring barely reaches them.

Climate science, epidemiology, and genomics were each transformed when measurement stopped being piecemeal and became part of the permanent infrastructure: standardized, continuous, and openly shared. Insect ecology remains in the pre-observatory phase. Despite its implications for agriculture, ecosystem resilience, and planetary stability, biodiversity decline is still inferred largely from fragmented local projects and uneven time series. The technological barriers involved are beginning to fall: computer vision, passive acoustics, DNA metabarcoding, automated imaging, environmental sequencing, and AI-assisted identification now make continental-scale monitoring technically plausible [8]. These modalities are not interchangeable, and none spans insect diversity alone. The plausible near-term approach is standardized Malaise-trap networks coupled with DNA metabarcoding, the only approach that currently delivers broad taxonomic coverage together with a biomass proxy at scale; automated imaging provides the continuous abundance time series that bulk sampling cannot, though largely for light-attracted macro-moths; and acoustic and environmental-DNA sensing add complementary coverage rather than serving as primary instruments. The point is architectural: an observatory for insects will be a deliberately heterogeneous, standardized network, not a single sensor. The remaining obstacles are institutional, political, and financial—biodiversity monitoring is still treated as fragmented short-term research rather than as core scientific infrastructure.

The agendas that shape biodiversity science, and the funding behind them, sit in the temperate Global North, while the diversity most needing monitoring lies in the Global South. Specimens, data, and analytical capacity continue to flow northward, leaving the countries richest in insect life with the least infrastructure to study their own biotas [9]. This compounds a deeper asymmetry: the drivers of decline—fossil-fuel emissions, intensive agriculture, outsized consumption—are disproportionately the responsibility of the world’s wealthiest, while the losses fall on the communities least able to absorb them. A credible global observatory therefore cannot be an extractive enterprise run from the North; it must be durable, locally rooted infrastructure, with sustained investment in scientific capacity where insect diversity is concentrated [9]. As in meteorology or infectious-disease surveillance, its value would come not from individual sites but from standardized measurements spread across climatic zones and sustained over decades, with particular emphasis on the tropics, where biodiversity is highest and monitoring density lowest. The goal is not merely to document decline but to identify where, when, and why insect communities are changing, and to provide an early warning system for ecological disruption. One caveat is decisive: metabarcoding and automated identification both fail without reference databases, and tropical reference libraries remain radically incomplete, so we cannot yet even name what we are losing.

Two conclusions follow. First, chemical transparency is a cheap measure that we need not wait for complete attribution to take. Continued emergency authorizations of persistent systemic insecticides are decisions made on the basis of incomplete but already concerning evidence, with the burden of proof pointing the wrong way. Establishing mandatory public reporting of agricultural pesticide use, including the seed-applied insecticides that dropped out of US national estimates after 2014 [6], would be a low-cost intervention that would substantially improve attribution and accountability. Second, the monitoring gap is fundamentally a problem of funding, policy, and infrastructure, not of technology. Sustained, locally led tropical monitoring is achievable, and standardized cross-site protocols such as the ForestGEO Arthropod Initiative show that network-level monitoring is within reach [10]. The limited tropical data we do have is not reassuring: the few long-term datasets generally report decline rather than stability [3], so the gap cannot be assumed to be benign. The question is whether governments and biodiversity frameworks will fund monitoring at the scale required. Protected areas embedded in chemically intensive landscapes will remain vulnerable, regardless; conservation policy cannot rely on reserve designation while leaving the surrounding landscape unchanged.

The defining question is no longer whether insect numbers are changing. It is whether human societies are willing to measure the living systems on which they depend before those systems reorganize beyond recovery. The next decade will determine whether insect decline remains a debate conducted through sparse datasets or becomes a globally monitored component of planetary environmental forecasting.
